# Swertiamarin fails to induce cytotoxicity in colon cancer cell lines: Evidence against a direct anticancer effect

**DOI:** 10.1371/journal.pone.0344653

**Published:** 2026-03-16

**Authors:** Ghada Khawaja, Aya Shoujaa, Youmna El-Orfali, Sonia Abou Najem, Michel J. Massaad

**Affiliations:** 1 Department of Biological Sciences, Faculty of Science, Beirut Arab University, Beirut, Lebanon; 2 Department of Experimental Pathology, Immunology and Microbiology, Faculty of Medicine, American University of Beirut, Beirut, Lebanon; 3 Health Sciences Division, Abu Dhabi Women’s College, Higher Colleges of Technology, Abu Dhabi, The United Arab Emirates; 4 Division of Pediatric Infectious Diseases, Department of Pediatrics and Adolescent Medicine, American University of Beirut Medical Center, Beirut, Lebanon; 5 Center for Infectious Diseases Research, American University of Beirut, Beirut, Lebanon; 6 Research Center of Excellence in Immunity and Infections, American University of Beirut, Beirut, Lebanon; Institute for Biological Research, University of Belgrade, SERBIA

## Abstract

**Background:**

Colorectal cancer (CRC) is the third most common cancer worldwide, with rising incidence linked to unhealthy lifestyle habits. Although 5-fluorouracil (5-FU) remains a standard therapy, its efficacy is often limited by resistance and adverse effects. These limitations have driven interest in plant-derived bioactive compounds as complementary or alternative therapies. This study investigated the potential anticancer effects of Swertiamarin (SWT), a seco-iridoid glycoside with reported anti-inflammatory and antioxidant properties.

**Methods:**

Three human CRC cell lines (HT-29, HCT-116, and Caco-2) and a non-cancerous (Vero E6) cell line were treated with SWT sourced from three independent suppliers to ensure reproducibility. Cell viability was evaluated using Trypan blue exclusion and MTT assays, while scratch assay and microscopic analyses were used to assess migration and morphological changes. The antioxidant capacity of SWT was measured using hydrogen peroxide scavenging assays, and its antimicrobial activity was tested against *Escherichia coli* (*E. coli*) and *Staphylococcus aureus* (*S. aureus*).

**Results:**

SWT exhibited minimal or no effect on cell proliferation across all cell lines, concentrations, and time points, while 5-FU significantly reduced cell viability. Co-treatment with SWT and 5-FU did not enhance cytotoxicity, indicating no synergistic effect. SWT also had no impact on cell migration or morphology. However, it demonstrated strong antioxidant activity comparable to ascorbic acid and displayed antimicrobial effects, transiently inhibiting *E. coli* and persistently suppressing *S. aureus*.

**Conclusion:**

SWT does not exert direct cytotoxic or antimigratory effects in CRC models but possesses potent antioxidant and antimicrobial activities. These findings suggest a possible chemopreventive or protective role for SWT rather than a therapeutic one. This study underscores the importance of rigorous and reproducible evaluation of pure natural products to accurately define their biological and therapeutic potential.

## Introduction

Colorectal cancer (CRC) is a malignancy that develops in the colon and rectum, primarily arising from the aberrant proliferation of glandular epithelial cells, with adenocarcinoma representing about 96% of cases worldwide [1]. Globally, CRC poses a major public health challenge, ranking the third most common type in terms of incidence and the second in terms of mortality in 2020, with an estimated 1.93 million new cases and 0.94 million deaths. It is predicted that the number of cases may rise to 3.2 million by 2040 [2].

The primary therapeutic intervention for CRC is chemotherapy, with 5-fluorouracil (5-FU) serving as the pivotal element of conventional treatment regimens [1,3]. However, its clinical utility is limited by systemic toxicity and the development of resistance. These challenges have prompted growing interest in natural products as adjunct therapies, given their potential to improve efficacy, reduce adverse effects, and exert synergistic effects with 5-FU [1,4–6].

Swertiamarin (SWT), a seco-iridoid glycoside derived from *Swertia* species, has been reported to exhibit diverse bioactivities, including anticancer, antioxidant, anti-inflammatory, and antimicrobial effects in various experimental models [7–11]. SWT has demonstrated anti-proliferative activity in two animal models of liver and gastric cancer [12,13], as well as in two studies on human hepatic [12,14], one study of cervical [15], and one study of neuroblastoma cancer cell lines [8]. Furthermore, SWT has been reported to induce morphological changes in cervical cancer (HeLa) cells, characterized by apoptotic features such as nuclear fragmentation and membrane rupture [15], and to reduce migration and invasion in hepatocellular carcinoma cells [12]. Mechanistically, studies indicate that SWT can suppress key oncogenic signaling pathways commonly over activated in CRC, including Wnt/β-catenin, PI3K/AKT, and RAS/ERK [12,15,16]. Furthermore, plant-extracted SWT has been reported to inhibit the proliferation of HT-29 colon cancer cells [9]. Despite these findings, none of the studies have evaluated the effect of pure, commercially available SWT across CRC cell lines with distinct molecular characteristics, such as HCT-116 and Caco-2, or examined its potential to synergize with standard chemotherapeutics like 5-FU.

SWT has demonstrated antibacterial activity against *Escherichia coli* (*E. coli*), *Staphylococcus aureus* (*S. aureus*), *Pseudomonas aeruginosa*, and *Bacillus subtilis* [11,17]. In addition, previous studies have shown that SWT enhances the activities of key antioxidant enzymes, such as glutathione reductase (GR), superoxide dismutase (SOD), glutathione peroxidase (GPx), glutathione-s-transferase (GST), and catalase (CAT), thereby alleviating oxidative stress in several *in vivo* rodent models [10,18–21]. Moreover, SWT reduces the production of reactive oxygen species (ROS) and lactate dehydrogenase (LDH), further supporting its protective role against oxidative damage [22]. However, in the present study, only the chemical antioxidant capacity of SWT was assessed using a hydrogen peroxide (H₂O₂) scavenging assay. These findings support further evaluation of SWT, particularly its potential antimicrobial and antioxidant properties.

Given the limitations of current CRC therapies and the reported biological properties of SWT, its direct effects in CRC models remain inconclusive. This study therefore aimed to systematically evaluate the biological activity of commercially sourced SWT across multiple CRC cell lines, including its cytotoxic and antiproliferative effects, impact on cell migration and morphology, and potential modulation of 5-FU response. SWT’s antimicrobial and chemical antioxidant properties were also assessed to confirm compound stability and bioactivity. The importance of this study lies in its rigorous and standardized approach, using three independently, high-purity sourced SWT preparations and multiple CRC cell lines with distinct molecular profiles. The findings provide a reproducible evidence base to determine whether SWT warrants further *in vivo* or clinical investigation as a complementary or chemopreventive agent.

## Materials and methods

### Cell lines, cell culture and drug preparation

Human colorectal adenocarcinoma cell lines (HCT-116, ATCC-CCL-247; HT-29, ATCC- HTB-38; and Caco-2, ATCC- HTB-37), the human hepatocellular carcinoma cell line (HepG2, ATCC- HB-8065), and the non-human, African green monkey kidney-derived cell line (Vero E6, ATCC- C1008) were cultured in Dulbecco’s Modified Eagle’s Medium (DMEM) (Corning, 10–013-CV) supplemented with 10% fetal bovine serum (FBS, BIOTECKNO, 10500064), 1% L-glutamine (Corning, 25–005-CI), and 1% penicillin/streptomycin (Corning, 30–002-CI). Cells were maintained at 37° C in a humidified incubator with 5% CO_2_ and were used at low passage numbers (passages 5–10) to minimize passage-related phenotypic and metabolic variability. 5-FU was purchased from (Cayman Chemical, 14416). SWT was purchased from MedChemExpress (HY-N0807), Cayman Chemical (27634), and Sigma-Aldrich (90957). For clarity, these preparations are hereafter referred to as SWT-α, SWT-β, and SWT-γ, respectively. The multiple sources of SWT were included to confirm the reproducibility of findings across different commercially available preparations, and to reduce the likelihood that the results are affected by supplier-specific impurities or formulation differences. Saponin was purchased from Sigma-Aldrich (SAE0073). Stock solutions of SWT (100 mg/mL) and 5-FU (53 mg/mL) were freshly prepared in dimethyl sulfoxide (DMSO) and further diluted with DMEM media to obtain the desired concentrations. The final DMSO concentration varied depending on the drug concentration, with a maximum of 0.4% v/v. Vehicle control groups containing equivalent DMSO concentrations were included for each treatment condition.

### Cell viability assays

#### Trypan blue exclusion assay.

Cell viability was assessed using the trypan blue exclusion method as described previously [23]. Briefly, HT-29, Caco-2, HCT-116 and Vero E6 cells were seeded in 96-well plates (6,500 cells per well). Cells were then treated with various concentrations of SWT-α, SWT-β, and SWT-γ (25–1000 µM) for 24, 48, and 72 h. At the indicated time points, cells were harvested, mixed with trypan blue solution (Sigma-Aldrich, T8154), and viable cells were quantified using a hemocytometer. All experiments were performed independently in triplicate, and results were expressed as the percentage of survival relative to vehicle-treated control (DMSO). For each treatment condition, a vehicle control containing the equivalent DMSO concentration was included. Data from treated groups were normalized to their corresponding vehicle controls to account for any solvent-related effects.

#### MTT assay.

Cell cytotoxicity was assessed using the 3-(4,5-dimethylthiazol-2-yl)-2,5-diphenyl tetrazolium bromide (MTT) assay as described previously [23]. Briefly, HT-29, Caco-2, HCT-116, Vero E6, and HepG2 cells were seeded in 96-well plates (6,500 cells per well). Cells were treated with various concentrations of SWT-α, SWT-β, and SWT-γ, 5-FU, or a combination of SWT-γ (1000 µM) with 5-FU (10–100 µM) for 24, 48, and 72 h. In selected experiments, SWT-γ treatment was preceded by a 5-minute pre-incubation with saponin (50 µg/mL) to transiently increase membrane permeability. At the indicated time points, MTT solution (Sigma-Aldrich, 11465007001) was added to each well to a final concentration of 0.5 mg/mL and incubated for 3 h at 37° C. The resulting formazan crystals were dissolved in isopropanol, and absorbance was measured at 595 nm using a microplate reader (Thermo Scientific MULTISKAN SkyHigh). All experiments were performed independently in triplicate, and results were expressed as the percentage of cytotoxicity relative to DMSO. For each treatment condition, a vehicle control containing the equivalent DMSO concentration was included. Data from treated groups were normalized to their corresponding vehicle controls to account for any solvent-related effects.

#### Cell morphology assay.

The morphology of CRC cells was evaluated following treatment with SWT-γ. HT-29, Caco-2, and HCT-116 cells were seeded in 96-well plates (6,500 cells per well). Cells were then treated with 1000 µM SWT-γ or an equivalent concentration of DMSO as a vehicle control. Cells were maintained under these conditions for 72 h without disturbance. At the end of the incubation period, cell morphology was examined and documented using light microscopy.

#### Scratch assay.

The migration capability of HT-29 colon cancer cells was assessed using a scratch assay as described previously [24]. Cells were seeded in 6-well plates at a density of 0.15 × 10^6^ cells/well and cultured until reaching 85%–95% confluence. A uniform scratch was created across the cell monolayer to generate a cell-free gap. After two washes with PBS, 1068 µM SWT-γ was added. Cell migration into the wound area was monitored at 12, 24, and 48 h, and images were captured using light microscopy. The wound area was quantified using ImageJ software.

#### Broth microdilution assay.

The antibacterial activity of SWT-γ and Amikacin was evaluated against *E. coli* (ATCC, 25922) and *S. aureus* (ATCC, 29213) using a broth microdilution assay. Standardized bacterial suspensions were prepared to match the turbidity of a 0.5 McFarland standard (~1 × 10⁸ CFU/mL) and diluted in Mueller-Hinton cation-adjusted broth (MHCAB; Sigma-Aldrich, 90922-500G) to obtain a working inoculum of 5 × 10⁶ CFU/mL. For the assay, 5 µL of the inoculum was added to 45 µL of MHCAB in each well of a sterile 96-well plate, resulting in a final concentration of 5 × 10⁵ CFU/mL per well. Serial two-fold dilutions of SWT-γ (3–3340 µM) and Amikacin (0.25–256 µg/mL) were prepared in MHCAB. Plates were incubated on a shaker at 160 rpm, and the absorbance of bacterial growth was measured at 600 nm after 16 and 24 h post-treatment using a microplate reader. Results were expressed as the percentage of bacterial growth inhibition relative to vehicle-treated controls.

#### Hydrogen peroxide scavenging assay.

The hydrogen peroxide (H₂O₂) scavenging activity of SWT-γ and ascorbic acid was evaluated as described previously [25]. Various concentrations of SWT-γ or ascorbic acid (65, 125, 250, 500, and 1000 µM) were prepared, and 1 mL of each was added to 2 mL of a 40 mM H₂O₂ solution in PBS (pH 7.4). The reaction mixtures were incubated for 10 minutes at room temperature. The absorbance of each sample was measured at 230 nm against a blank solution containing PBS and the respective compound without H₂O₂ using a spectrophotometer. The experiment was performed in triplicate, and results were expressed as the percentage of H₂O₂ scavenging activity relative to the control.

### Statistical analysis

Data were analyzed using GraphPad prism software (Ver. 9.3.1). Statistical significance between groups was assessed using two-way ANOVA and student’s t test. Statistical significance is indicated as follows: p < 0.0001****, p < 0.001***, p < 0.05*, and non-significant differences as p > 0.05.

## Results

### SWT-α, SWT-β, and SWT-γ do not affect cell viability in CRC and vero E6 cells across a broad concentration range

**Fig 1:**
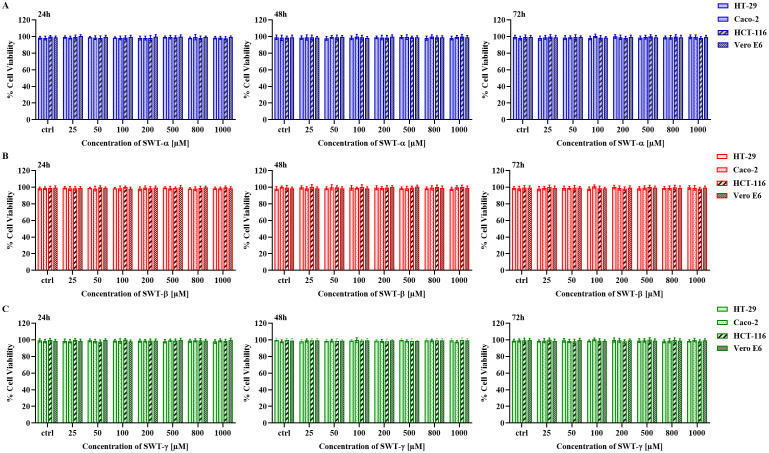
Treatment with SWT-α, SWT-β, and SWT-γ does not affect the viability of HT-29, Caco-2, HCT-116, or Vero E6 cells across 24–72 h. HT-29, Caco-2, HCT-116 and Vero E6 were treated with SWT-α (A), SWT-β (B), and SWT-γ (C) at concentrations ranging from 25–1000 µM for 24, 48, and 72 h. Cell viability was assessed using the trypan blue exclusion assay and expressed as the percentage of viable cells relative to DMSO-treated control (Ctrl). No significant differences were observed among the tested concentrations at 24, 48 and 72 h for each cell line, compared with the DMSO control, using all SWT sources (p > 0.05). Data are presented as mean ± SD from three independent experiments, each performed in triplicate (n = 3).

To assess the effect of SWT from three different sources on cell viability in cancerous and non-cancerous cell lines, HT-29, Caco-2, HCT-116 and Vero E6 cells were treated with SWT-α (Fig 1A), SWT-β (Fig 1B), and SWT-γ (Fig 1C), at concentrations ranging from 25 to 1000 µM for 24, 48, and 72 h. Cell viability was assessed using the trypan blue exclusion method (Fig 1). HT-29 cells maintained high viability under all treatment conditions, with values of 99.63 ± 2.28% (SWT-α), 99.6 ± 2.26% (SWT-β), and 99.03 ± 1.68% (SWT-γ), compared with controls of 99.4 ± 2.12%, 98.83 ± 1.83%, and 98.72 ± 1.4% (Fig 1A–1C). Similarly, Caco-2, HCT-116, and Vero E6 cells exhibited consistently high viability (>98%) across all SWT sources, with no statistically significant differences relative to DMSO control. Overall, SWT from all three sources maintained cell viability under the tested conditions.

### SWT-α, SWT-β, and SWT-γ fail to induce cytotoxicity in CRC and vero E6 cells across time and dose ranges

**Fig 2:**
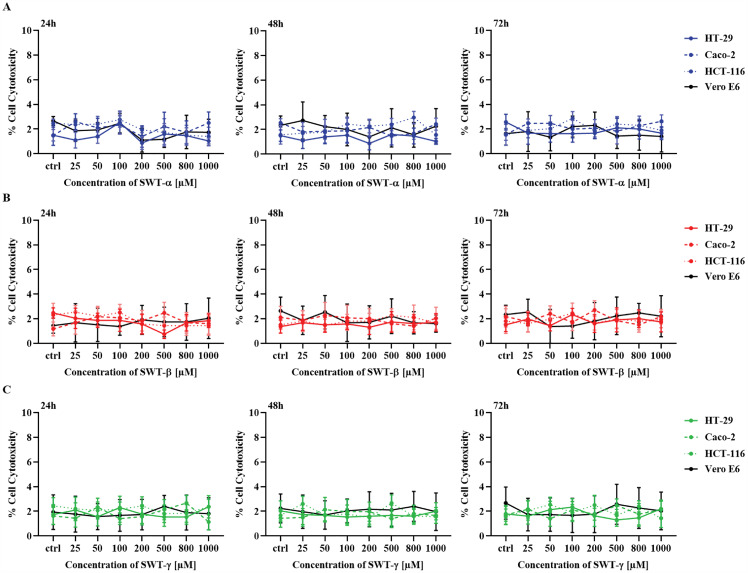
SWT-α, SWT-β, and SWT-γ fail to induce cytotoxicity in HT-29, Caco-2, HCT-116, and Vero E6 cells following 24, 48, and 72 hours of treatment. HT-29, Caco-2, HCT-116 and Vero E6 were treated with SWT-α (A), SWT-β (B), and SWT-γ (C) at concentrations ranging from 25–1000 µM for 24, 48, and 72 h. Cytotoxicity was evaluated using the MTT assay, and values were expressed as % cytotoxicity relative to DMSO-treated control (Ctrl). No significant differences were observed among the tested concentrations at 24, 48 and 72 h for each cell line, compared with the DMSO control, using all SWT sources (p > 0.05). Data are presented as mean ± SD from three independent experiments, each performed in triplicate (n = 3).

Given that trypan blue assesses membrane integrity but may not capture minor metabolic changes, we further examined SWT’s cytotoxic potential using the MTT assay, which provides a more sensitive measure of cell viability through metabolic activity (Fig 2). HT-29, Caco-2, HCT-116 and non-cancerous Vero E6 cells were treated with SWT-α (Fig 2A), SWT-β (Fig 2B), and SWT-γ (Fig 2C), at concentrations up to 1000 µM for 24, 48, and 72 hours. HT-29 cells treated with 1000 µM exhibited minimal cytotoxicity at all-time points, with values of 1.03 ± 0.4% (SWT-α), 1.73 ± 1.40% (SWT-β) and 2.2 ± 1.23% (SWT-γ), compared with controls of 1.5 ± 0.8%, 1.5 ± 0.96%, and 1.8 ± 1.05% (Fig 2A–2C). Similarly, Caco-2, HCT-116, and Vero E6 cells were minimally affected, with cytotoxicity consistently below 3% across all SWT sources and time points. Overall, SWT treatment at concentrations up to 1000 µM did not induce cytotoxicity in any tested cell line over time, and no statistically significant differences were observed compared with controls.

### 5-FU exhibits time- and dose-dependent cytotoxicity in CRC cells

**Fig 3:**
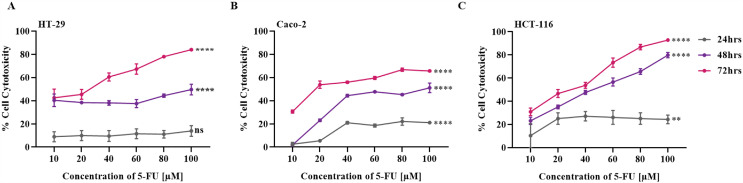
5-FU induce cytotoxicity in HT-29, Caco-2 and HCT-116 cells following 24, 48, and 72 hours of treatment. HT-29 (A), Caco-2 (B) and HCT-116 (C) were treated with increasing concentrations of 5-FU (10–100 μM) for 24, 48, and 72 **h.** Cytotoxicity was evaluated using the MTT assay, and values were expressed as % cytotoxicity relative to untreated controls. Statistical significance was observed between treated (5-FU) and control (DMSO) cells as follows: HT-29, 24 h (p > 0.05) and 48–72 h (****p < 0.0001); Caco-2, 24 h (***p ≤ 0.001) 48-–72 h (****p < 0.0001); HCT116, 24 h (p > 0.05) and 48–72 h (****p < 0.0001). Data are presented as mean ± SD (n = 3).

Since SWT from all three sources neither reduced cell viability nor induced cytotoxicity, we next assessed the sensitivity of CRC cells to chemotherapeutic challenge using 5-FU, the standard agent used in CRC treatment. This was done to confirm that the CRC cell models were responsive to chemotherapy rather than resistant. HT-29, Caco-2, and HCT-116 cells were treated with increasing concentrations of 5-FU for 24, 48, and 72 hours (Fig 3A–3C). In HT-29 cells, cytotoxicity remained minimal at 24 h (<15% at 100 μM) and moderate at 48 h (~40–45%), but increased sharply at 72 h in a dose-dependent manner, reaching ~80% at 100 μM, with an IC₅₀ of 23.79 ± 8.483μM at 72 h. Caco-2 cells exhibited the least sensitivity, with cytotoxicity below 20% at 24 h, ~ 45% at 48 h, and up to 65% at 72 h, corresponding to an IC₅₀ of 26.54 ± 5.57 μM at 72 h. In contrast, HCT-116 cells were the most sensitive, showing significant cytotoxicity as early as 24 h (~20–25% at 20–40 μM) and reaching ~70% at 48 h and ~85–90% at 72 h at 100 μM, with IC₅₀ values of 38.74 ± 5.22 μM (48 h) and 23.44 ± 7.79 μM (72 h). Collectively, these findings demonstrate a dose- and time-dependent cytotoxic effect of 5-FU across all tested CRC cell lines, with HCT-116 and HT-29 being the most responsive, while Caco-2 exhibited the highest resistance at all time points. These results confirm that the CRC cells are viable and remain sensitive to chemotherapeutic challenge, validating their suitability for subsequent experiments.

### Combined treatment with SWT-γ and 5-FU fails to increase cytotoxicity in HT-29 cells

**Fig 4:**
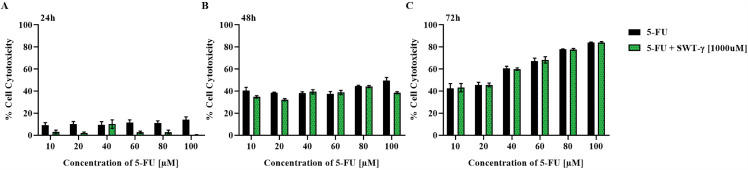
SWT-γ fails to potentiate the cytotoxic effect of 5-FU in HT-29 cells over 24–72 h. HT-29 cells were treated with 5-FU, alone or with 1000 µM SWT, for 24 h **(A)**, 48 h **(B)** and 72 h **(C)**. Cytotoxicity under different treatment conditions was assessed using the MTT assay. Values are expressed as percent cytotoxicity relative to untreated controls. No statistically significant differences were observed between the combination treatment and 5-FU alone at any time point (P > 0.05). Data are presented as mean ± SD (n = 3).

Although SWT-γ alone did not exhibit cytotoxic effects on three different CRC cell lines over time, 5-FU demonstrated significant, dose- and time-dependent cytotoxicity at all tested time points. Based on this, we hypothesized that combining SWT-γ with 5-FU might enhance the cytotoxic effect of 5-FU or produce a synergistic response. HT-29 cells were treated with 5-FU at concentrations ranging from 10 to 100 µM, with or without 1000 µM of SWT-γ, for 24, 48, and 72 h. After 24 h, the combination treatment resulted in 0.15 ± 0.93% cytotoxicity, which was lower than the 13.93 ± 6.57% observed with 100 µM 5-FU only (Fig 4A). At 48 h, the combination treatment produced 38.52 ± 2.38% cytotoxicity, compared to 49.62 ± 6.48% for 5-FU alone (Fig 4B). After 72 h, cytotoxicity was similar between the combination and 5-FU alone (84 ± 1.7% vs. 84 ± 0.8%, Fig 4C). These results indicate that SWT-γ failed to potentiate the cytotoxic effect of 5-FU, as the combination treatment did not produce higher cytotoxicity compared to 5-FU alone across all tested concentrations and time points (p > 0.05).

### SWT-γ does not elicit cytotoxic effects following transient membrane permeabilization or serum deprivation

**Fig 5:**
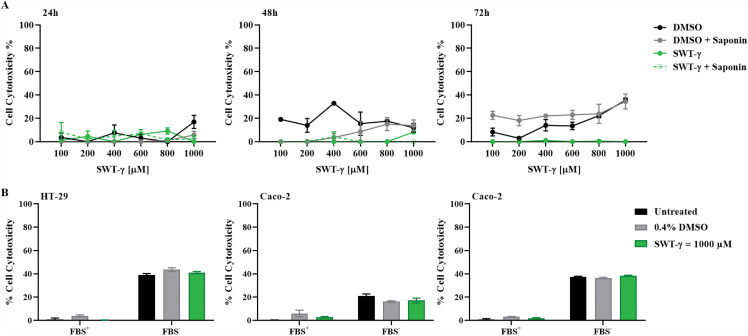
Transient membrane permeabilization or serum deprivation does not restore SWT-γ Cytotoxicity. (A) HT-(A) HT-29 cells were treated with increasing concentrations of SWT-γ or DMSO for 24–72 h, with or without saponin pre-permeabilization (50 µg/mL). Cells were treated with SWT (100–1000 µM) or matched vehicle controls containing equivalent DMSO concentrations (maximum 0.4% v/v) in the presence of saponin; data were normalized to the corresponding vehicle controls. (B) HT-29, HCT-116, and Caco-2 cells were exposed to 1000 µM SWT-γ, DMSO, or serum-free media for 24 h. Cytotoxicity was assessed by MTT assay, and no significant differences were observed compared with controls (p > 0.05). Data are presented as mean ± SD (n=3).

Since SWT-γ showed no intrinsic cytotoxicity and failed to enhance 5-FU–mediated effects, we next investigated whether its inactivity could be attributed to experimental limitations, such as limited cellular uptake or serum-dependent effects [26,27]. To assess the potential influence of limited uptake, HT-29 cells were pretreated with saponin (50 µg/mL) to transiently increase membrane permeability prior to exposure to SWT-γ or vehicle control (DMSO). MTT assays performed at 24–72 h revealed no cytotoxic effects, indicating that insufficient uptake did not account for the inactivity (Fig 5A). Furthermore, to determine whether serum components in FBS interfered with SWT-γ activity, HT-29, HCT-116, and Caco-2 cells were treated with 1000 μM SWT-γ in serum-free media for 24 h (Fig 5B). Under these conditions, SWT-γ did not alter or enhance cytotoxicity compared with DMSO or serum-free controls, and this result was consistent across all three cell lines. Together, these findings indicate that the inactivity of SWT-γ is intrinsic rather than due to experimental limitations.

### HepG2 cells are resistant to SWT-γ but responsive to 5-FU

**Fig 6:**
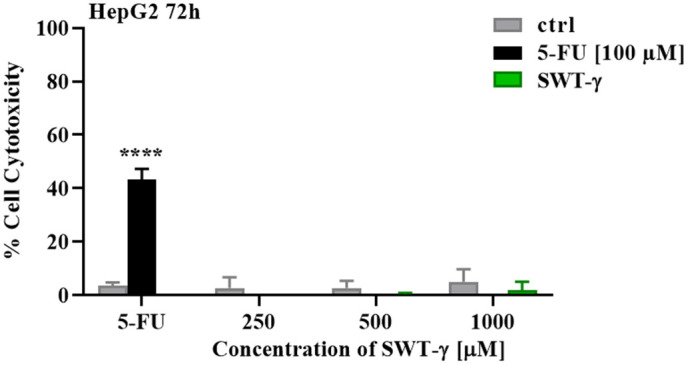
SWT-γ does not induce cytotoxicity in HepG2 cells. HepG2 cells were treated for 72 h with various concentrations of SWT-γ (267–1068µM), 5-FU (100 µM), or DMSO (cntrl). Cytotoxicity was assessed by MTT assay and expressed as percent relative to untreated controls. SWT-γ did not induce significant cytotoxicity (P > 0.05), whereas 5-FU caused robust cytotoxicity (****P < 0.0001, student’s t test) Data are presented as mean ± SD (n = 3).

Previous studies have reported that SWT-γ exhibits cytotoxicity against HepG2 hepatocellular carcinoma cells, with an IC50 of approximately 234.97 µM [12,14]. To assess the cytotoxic potential of SWT-γ, HepG2 cells were treated with SWT-γ (267, 534, and 1068 µM) or 100 µM 5-FU for 72 h. Contrary to previous reports, SWT-γ did not induce cytotoxicity, even at the highest concentration (1068 µM; Fig. 6). In contrast, 100 µM 5-FU induced 43.31 ± 3.99% cytotoxicity, confirming that the HepG2 cells remained responsive to cytotoxic agents (Fig. 6). These results indicate that, under the conditions tested, SWT-γ does not exert cytotoxic effects in HepG2 cells, highlighting a discrepancy with earlier studies and confirming the efficacy of 5-FU as a positive control.

### Absence of morphological alterations in CRC cells exposed to SWT-γ

**Fig 7:**
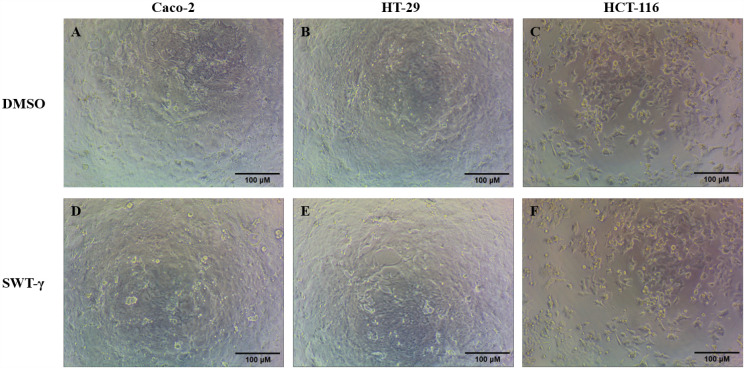
Morphological assessment of CRC cells following SWT-γ treatment. Caco-2, HT-29, and HCT-116 were either treated with DMSO (A–C) or with 1000 µM of SWT-γ **(D–F)**. Images were captured using a 10 × objective lens and are shown with a scale bar of 100 µm.

SWT has been reported to induce dose-dependent cytotoxicity in HeLa cells, characterized by apoptotic features such as nuclear fragmentation and membrane rupture [15]. To investigate the effect of SWT-γ on CRC cell lines, HT-29, Caco-2, and HCT-116 cells were treated with 1000 µM SWT-γ or an equivalent concentration of DMSO as a vehicle control and incubated for 72 h (Fig. 7). Cell morphology was assessed using light microscopy, and no apparent differences were observed between SWT-γ–treated and control cells at this concentration and time point. These findings suggest that SWT-γ does not exert observable cytotoxic effects on CRC cell lines at a concentration of 1000 µM within 72 h.

### SWT-γ treatment does not alter cell migration in HT-29 cells

**Fig 8:**
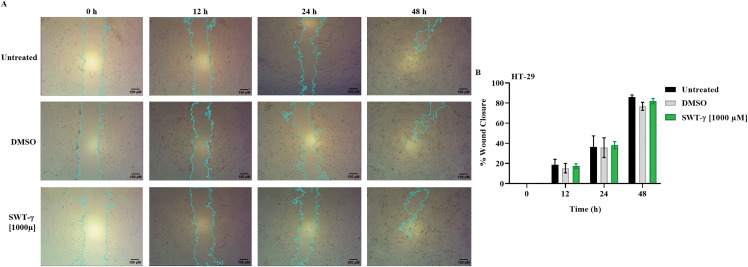
Wound closure of HT-29 cells over time following SWT-γ treatment. **(A)** Representative light microscopy images of scratches in HT-29 cells at 0, 12, 24, and 48 h after being left untreated or treated with DMSO or 1000 µM SWT-γ. Images were captured using a 4x objective lens. Scale bar = 100 µm. **(B)** Quantification of wound closure over time, expressed as percentage closure. No statistically significant differences were observed between the untreated or treated with DMSO or 1000 µM SWT-γ at any time point (P > 0.05). Data are presented as mean ± SD (n = 3).

Scratch assay is commonly used to assess drug effects on cell migration, and previous studies have reported that SWT reduced migration and invasion in hepatocellular carcinoma cells [12]. To evaluate its effect on colon cancer cells, HT-29 cells were scratched and either left untreated or treated with DMSO or 1000 µM SWT-γ (Fig 8). Scratch closure was monitored at 0, 12, 24, and 48 h using light microscopy (Fig 8A) and quantified as percentage closure using ImageJ software (Fig 8B). At 12 h, wound closure reached 18.72 ± 5.39% in untreated cells, 17.41 ± 2.27% in SWT-γ–treated cells, and 15.22 ± 4.63% in DMSO-treated cells. By 24 h, closure increased to 36.47 ± 10.87%, 38.18 ± 3.52%, and 35.67 ± 1.8% in untreated, SWT-γ, and DMSO-treated cells, respectively. At 48 h, closure was highest in untreated cells (85.89 ± 2.17%), followed by SWT-γ–treated cells (81.85 ± 2.89%) and DMSO-treated cells (76.79 ± 4.02%). The similarity between SWT-γ–treated and DMSO-treated cells across all time points indicates that SWT-γ does not impair wound healing in HT-29 cells.

### SWT-γ demonstrates substantial H₂O₂ scavenging but limited antibacterial efficacy

Since SWT showed no cytotoxic effects and did not impair HT-29 cell migration, its potential bioactive properties were explored to determine if the preparation in hand was bioactive. Specifically, the antimicrobial activity of SWT was assessed against *E. coli* and *S. aureus*, and its antioxidant potential was evaluated using H₂O₂ scavenging assays. These experiments aimed to characterize the functional bioactivities of SWT beyond cytotoxicity and migration inhibition.

**Fig 9:**
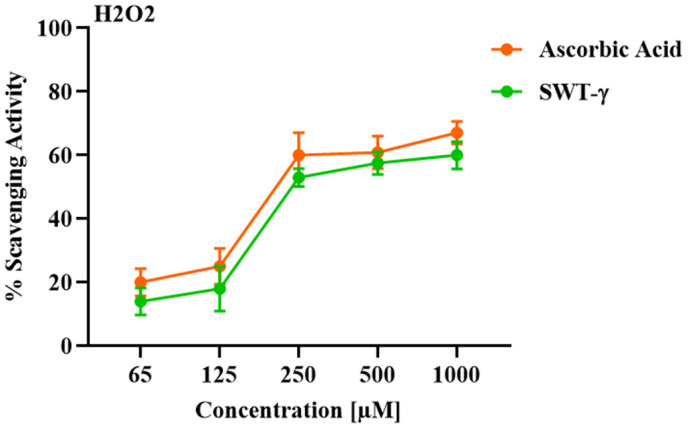
Hydrogen peroxide (H₂O₂) scavenging activity of Ascorbic Acid and SWT-γ. No significant difference was observed between ascorbic acid and SWT-γ (p > 0.05). Data are presented as mean ± SD (n = 3).

The H₂O₂ scavenging activity of SWT-γ was evaluated and compared to the well-known antioxidant ascorbic acid across a range of concentrations (65, 125, 250, 500, and 1000 µM). Both compounds demonstrated a clear concentration-dependent increase in scavenging activity (Fig 9). At the lowest concentrations tested (65 and 125 µM), ascorbic acid exhibited slightly higher scavenging activity, reaching approximately 20% and 25%, respectively, whereas SWT-γ scavenged ~15% and 18% of H₂O₂ at the same concentrations. At 250 µM, a marked increase in activity was observed for both compounds: ascorbic acid reached ~60%, while SWT-γ achieved ~53%. At higher concentrations (500 and 1000 µM), the scavenging activities of both compounds approached a plateau. Ascorbic acid showed maximal activity of ~67%, whereas SWT-γ reached ~60%. These results indicate that while SWT-γ exhibited slightly lower efficacy compared to the positive control, it nonetheless demonstrated substantial H₂O₂ scavenging capability in a dose-dependent manner.

Amikacin exhibited strong dose- and time-dependent bactericidal activity against both *E. coli* and *S. aureus*, achieving over 99% inhibition at concentrations ≥2 µg/mL at 16 and 24 hours (Table 1). At 16 hours, inhibition of *E. coli* increased from ~20% at 0.5 µg/mL to 92% at 16 µg/mL, whereas *S. aureus* showed slightly lower inhibition across all concentrations, ranging from ~15% at 0.5 µg/mL to 87% at 16 µg/mL. By 24 hours, inhibition further increased, reaching near-complete levels (~98%) for *E. coli* and ~94% for *S. aureus*, confirming the rapid and robust bactericidal effect of Amikacin.

**Table 1:** % Inhibition of *E. coli* and *S. aureus* after treatment with Amikacin at 16 h and 24h.

	% Inhibition (16h)		% Inhibition (24h)	
Amikacin [µg/mL]	*E. coli*	*S. aureus*	*E. coli*	*S. aureus*
0	0.00 ± 0.00	0.00 ± 0.00	0.00 ± 0.00	0.00 ± 0.00
0.25	3.30 ± 1.27	14.33 ± 13.04	0.00 ± 0.00	0.00 ± 0.00
0.5	36.99 ± 0.13	31.58 ± 11.13	0.00 ± 0.00	7.15 ± 0.75
1	93.69 ± 2.93	99.97 ± 0.69	93.50 ± 1.55	100.69 ± 0.08
2	99.54 ± 0.38	100.10 ± 0.16	100.98 ± 0.21	100.64 ± 0.06
4	99.60 ± 0.06	100.07 ± 0.18	100.98 ± 0.29	100.69 ± 0.07
8	98.44 ± 1.78	99.86 ± 0.24	99.64 ± 2.07	100.12 ± 0.33
16	99.31 ± 0.13	99.87 ± 0.26	100.67 ± 0.09	100.46 ± 0.08
32	99.39 ± 0.07	100.06 ± 0.08	100.87 ± 0.04	100.54 ± 0.23
64	99.50 ± 0.09	100.09 ± 0.10	100.85 ± 0.16	100.69 ± 0.12
128	99.11 ± 0.33	100.25 ± 0.02	100.84 ± 0.01	100.71 ± 0.12
256	99.60 ± 0.14	100.29 ± 0.16	100.63 ± 0.67	100.58 ± 0.26

In comparison, SWT-γ demonstrated moderate antibacterial activity. Against *E. coli*, inhibition was transient, starting at 26 µM (4.61 ± 7.41%) and peaking at 835 µM (23.92 ± 6.25%) at 16 hours, but was completely diminished by 24 hours (Table 2). *S. aureus* was less sensitive at lower concentrations, with inhibition beginning at 835 µM (4.17 ± 0.90%) at 16 hours and reaching 18.89 ± 3.32% at 3340 µM, while the effect became more pronounced at 24 hours, achieving 45.13 ± 1.8% inhibition at the highest concentration.

**Table 2:** % Inhibition of *E. coli* and *S. aureus* after treatment with SWT-γ at 16 h and 24h.

	% Inhibition (16h)		% Inhibition (24h)	
SWT-γ [µM]	*E. coli*	*S. aureus*	*E. coli*	*S. aureus*
0	0.00 ± 0.00	0.00 ± 0.00	0.00 ± 0.00	0.00 ± 0.00
3	0.00 ± 0.00	0.00 ± 0.00	0.00 ± 0.00	0.00 ± 0.00
7	2.54 ± 7.54	0.00 ± 0.00	0.00 ± 0.00	0.00 ± 0.00
13	−2.29 ± 7.45	0.00 ± 0.00	0.00 ± 0.00	0.00 ± 0.00
26	4.61 ± 7.41	0.00 ± 0.00	0.00 ± 0.00	0.00 ± 0.00
52	12.07 ± 13.76	0.00 ± 0.00	0.00 ± 0.00	0.00 ± 0.00
104	19.23 ± 22.14	0.00 ± 0.00	0.00 ± 0.00	0.00 ± 0.00
209	10.27 ± 11.49	0.00 ± 0.00	0.00 ± 0.00	0.00 ± 0.00
417	23.73 ± 6.28	0.00 ± 0.00	0.00 ± 0.00	0.00 ± 0.00
835	23.92 ± 6.25	4.17 ± 0.90	0.00 ± 0.00	0.00 ± 0.00
1670	19.87 ± 3.79	13.39 ± 4.89	0.00 ± 0.00	0.00 ± 0.00
3340	20.74 ± 4.81	18.89 ± 3.32	0.00 ± 0.00	45.126 ± 1.8

Overall, while SWT-γ exhibited modest antibacterial activity compared to Amikacin, particularly against *E. coli*. *S. aureu*s appeared more susceptible to SWT-γ at higher concentrations and longer exposure, suggesting that SWT-γ exhibits selective, time-dependent antibacterial effects that are weaker but sustained relative to the rapid, near-complete inhibition achieved by the positive control, Amikacin.

## Discussion

This study provides clear evidence that SWT, despite previous reports of anticancer potential, does not exhibit cytotoxic, antiproliferative, or antimigratory activity against CRC cell lines. Using three molecularly distinct CRC models (HT-29, Caco-2, and HCT-116), along with a non-cancerous Vero E6 cell line, and three independently sourced SWT preparations (SWT-α, SWT-β, SWT-γ), we observed negligible cytotoxic effects even at concentrations up to 1000 µM. Moreover, SWT did not enhance the activity of the standard chemotherapeutic agent 5-FU, indicating a lack of synergistic interaction. Instead, SWT displayed strong antioxidant and modest antibacterial properties, suggesting that its biological activity may be more protective than cytotoxic in nature.

Given the limitations of 5-FU-based chemotherapy in treatment of CRC [1,28], and the growing interest in natural compounds as complementary agents with lower toxicity [1,4], our results provide important clarification regarding the actual role of SWT and its therapeutic potential.

Previous studies have reported SWT-mediated cytotoxicity in several cancer models, including HT-29 and HepG2 cells [7–9,12,15]. For instance, SWT extracted from *Enicostemma axillare* demonstrated antiproliferative activity in HT-29 cells with IC₅₀ values between 133.5 and 267.1 µM depending on the assay used [9]. Yet, these results were based on plant-derived extracts with variable purity, and no comparisons were made across distinct CRC cell lines [9]. By contrast, our results using pure, commercially sourced SWT found no cytotoxic activity in HT-29, Caco-2, HCT-116, or Vero E6 cells, even under optimized culture conditions. As part of the experimental design, the Vero E6 cell line was included as a general non-tumorigenic reference model commonly employed in preliminary cytotoxicity screening. We acknowledge, however, that Vero E6 cells originate from non-human primate kidney epithelium and therefore do not represent an ideal non-cancerous model for direct biological comparison with human colorectal cancer cell lines. Species- and tissue-specific differences may limit the interpretability of such comparisons, and the absence of SWT-induced cytotoxicity in Vero E6 cells should therefore be interpreted as indicative of low general cytotoxicity rather than tissue-specific selectivity.

The discrepancy between our findings and earlier reports may reflect methodological differences. Several prior studies relied on plant-derived SWT without rigorous purity validation, raising the possibility that minor co-occurring phytochemicals contributed to the reported anticancer effects. Although chromatographic separation and NMR analyses support compound identity, they do not definitively establish chemical purity, and the absence of confirmatory methods such as HPLC/UPLC or LC–MS may allow low-level impurities to remain undetected [9]. In contrast, we evaluated three independently sourced SWT preparations with certified purity (≥99%) across multiple CRC cell lines and complementary functional assays, and included appropriate vehicle controls, thereby minimizing batch-related and experimental variability. Additional factors, including differences in cell models, seeding density, solvent concentration, and experimental design, may further contribute to divergent outcomes [15,29]. Collectively, these considerations support our findings that SWT lacks intrinsic anticancer activity in CRC models under well-controlled conditions.

Interestingly, recent evidence also supports a cytoprotective rather than cytotoxic role for SWT [30]. In a Crohn’s disease-like colitis model, SWT protected Caco-2 cells from TNF-α-induced apoptosis by suppressing pro-apoptotic proteins (cleaved caspase-3, Bax) and upregulating the anti-apoptotic protein Bcl-2 [30]. This mechanistic evidence aligns with our findings and further supports that SWT may contribute to cellular protection rather than induce cellular death.

Given the absence of intrinsic cytotoxicity, we next investigated whether SWT-γ could enhance the anticancer efficacy of 5-FU, a common approach used to potentiate chemotherapy [31]. Combining 5-FU with natural compounds has been extensively investigated as a strategy to enhance treatment efficacy, mitigate drug resistance, and reduce toxicity by enabling lower effective doses [6,32]. Based on this rationale, we hypothesized that SWT-γ might synergize with 5-FU to improve its antiproliferative activity against CRC cells. However, while 5-FU alone significantly inhibited cell proliferation, its combination with SWT-γ did not produce any synergistic or additive effects. To our knowledge, this is the first study to assess the combined action of SWT and 5-FU in colorectal cancer.

To rule out possible experimental artifacts, we further examined factors that could affect SWT bioavailability, including serum binding and limited membrane permeability. In cell culture, fetal bovine serum (FBS) contains bovine serum albumin, which can similarly sequester compounds and diminish their biological effects—a phenomenon previously observed for natural products like thymoquinone in HCT-116 cells [33]. Another important determinant of cytotoxic potential is cellular uptake, as compounds with low permeability may be restricted by the plasma membrane, limiting intracellular accumulation and subsequent target engagement [34]. However, neither serum deprivation nor transient membrane permeabilization with saponin restored SWT’s cytotoxic potential, suggesting that the compound’s inactivity is intrinsic rather than due to experimental constraints.

Extending these observations to liver cancer, we found that SWT-γ exhibited no cytotoxic effect on HepG2 cells, even at high concentrations. This finding is in line with a recent report showing that treatment with 1000 µM SWT for 48 hours did not affect HepG2 cell viability [35]. In contrast, earlier studies reported significant cytotoxic activity against HepG2 cells, with IC₅₀ values ranging from 234.97 to 277.06 µM when using SWT-γ obtained from Sigma-Aldrich [12,14]. In the present study, HepG2 cells were used at low passage numbers (P5–P10), a range known to preserve metabolic stability, proliferative capacity, and physiological responsiveness. By contrast, high-passage HepG2 cells are known to undergo metabolic reprogramming, including reduced proliferation and increased sensitivity to stress-induced cell death [36], factors that can significantly influence outcomes in metabolism-based viability assays. Although passage numbers were not reported in earlier studies, such passage-related metabolic variability may therefore explain the lower IC₅₀ values previously reported and the discrepancies among studies. Taken together, our results align with recent evidence indicating a lack of direct cytotoxic activity of SWT in HepG2 cells and highlight the importance of considering cell passage–related metabolic state when interpreting *in vitro* anticancer effects.

Morphological assessment and scratch assays provided additional evidence against SWT-induced cytotoxicity or antimigratory effects. Drug-induced morphological changes often provide important insights into drug efficacy, toxicity, and underlying mechanisms of action, and previous studies reported apoptotic-like features [15] and reduced migration following SWT treatment at concentrations ranging from 20 to 100 µg/mL [12]. In one report, SWT of unspecified origin exhibited dose-dependent cytotoxic effects on HeLa cells at concentrations ranging from 10 to 80 µM, characterized by apoptotic morphological features such as nuclear fragmentation and membrane rupture [15]. In contrast, treatment with SWT-γ in our study did not induce any noticeable morphological changes in colon cancer cell lines, further supporting the absence of cytotoxic activity observed in our viability assays. Similarly, SWT-γ had no effect on the migratory behavior of HT-29 cells, as wound closure occurred at rates comparable to untreated controls. While SWT has been reported to reduce migration and invasion in hepatocellular carcinoma cells [12], our findings indicate that it does not exert antimigratory effects in colon cancer models. Together, these results reinforce the conclusion that SWT’s anticancer activity is minimal or absent.

Nevertheless, SWT retained strong antioxidant capacity, achieving ~60% H₂O₂ scavenging activity comparable to ascorbic acid, and exhibited mild antibacterial effects, particularly against *S. aureus*. These findings confirm that the SWT preparations used in this study are biologically active under our experimental conditions, and align with reports attributing SWT’s benefits primarily to its antioxidant and antimicrobial actions [11,17,37]. Importantly, the antibacterial assays were included as part of a broader pharmacological characterization and served as a functional validation of compound stability and bioactivity following preparation and dissolution, rather than a direct measurement of SWT direct antibacterial potential. Demonstrating measurable antibacterial activity in an independent biological system supports the conclusion that the absence of cytotoxic, antiproliferative, or antimigratory effects in CRC models reflects the intrinsic inactivity of SWT in this context, rather than compound degradation or loss of biological function.

In summary, this study provides a systematic and comparative evaluation demonstrating that SWT lacks direct cytotoxic, antiproliferative, or antimigratory effects in colorectal and liver cancer cell models, even when tested across multiple independently sourced preparations, cancer cell lines with distinct molecular profiles, and complementary assay conditions. While both protective and cytotoxic effects of SWT have been reported previously in the literature, these findings were often derived from heterogeneous experimental systems or preparations with insufficiently defined purity. By generating robust and reproducible negative evidence under well-controlled conditions, the present study clarifies an important knowledge gap and indicates that SWT’s biological profile is more consistent with a cytoprotective, antioxidant compound that supports cellular integrity rather than inducing cancer cell death. These findings underscore the importance of rigorous validation of pure natural products across multiple experimental models before attributing anticancer properties and suggest that the therapeutic relevance of SWT may lie primarily in protective or preventive applications rather than in direct anticancer activity.

## Supporting information

S1 File S1A Fig. (PZFX)

S2 File S1B Fig. (PZFX)

S3 File S1C Fig. (PZFX)

S4 File S2A Fig. (PZFX)

S5 File S2B Fig. (PZFX)

S6 File S2C Fig. (PZFX)

S7 File S3A Fig. (PZFX)

S8 File S3B Fig. (PZFX)

S9 File S3C Fig. (PZFX)

S10 File S4A Fig. (PZFX)

S11 File S4B Fig. (PZFX)

S12 File S4C Fig. (PZFX)

S13 File S5A Fig. (PZFX)

S14 File S5B Fig. (PZFX)

S15 File S6 Fig. (PZFX)

S8 File S8 Fig. (PZFX)

S8 File S9 Fig. (PZFX)
